# *Wolbachia* strain *w*MelM disrupts egg retention by *Aedes aegypti* females prevented from ovipositing

**DOI:** 10.1128/aem.01491-24

**Published:** 2024-12-04

**Authors:** Perran A. Ross, Ella Yeatman, Mel S. Berran, Xinyue Gu, Ary A. Hoffmann, Belinda van Heerwaarden

**Affiliations:** 1 Pest and Environmental Adaptation Research Group, School of BioSciences, Bio21 Molecular Science and Biotechnology Institute, The University of Melbourne 195117 , Parkville, Australia; UMR Processus Infectieux en Milieu Insulaire Tropical, Ste. Clotilde, France

**Keywords:** *Wolbachia*, *Aedes aegypti*, egg retention, *w*MelM

## Abstract

*Aedes aegypti* mosquitoes are well adapted to dry climates and can retain their eggs for extended periods in the absence of suitable habitat. *Wolbachia* strains transferred from other insects to mosquitoes can be released to combat dengue transmission by blocking virus replication and spreading through populations, but host fitness costs imposed by *Wolbachia*, particularly under some environments, can impede spread. We, therefore, assessed the impact of two *Wolbachia* strains being released for dengue control (*w*AlbB and *w*MelM) on fecundity and egg viability following extended egg retention (up to 24 days) under laboratory conditions. Egg viability following retention decreased to a greater extent in females carrying *w*MelM compared to uninfected or *w*AlbB females. Fertility fully recovered in uninfected females following a second blood meal after laying retained eggs, while *w*MelM females experienced only partial recovery. Effects of *w*MelM on egg retention were similar regardless of whether females were crossed to uninfected or *w*MelM males, suggesting that fitness costs were triggered by *Wolbachia* presence in females. The fecundity and hatch proportions of eggs of *w*MelM females declined with age, regardless of whether females used stored sperm or were recently inseminated. Costs of some *Wolbachia* strains during egg retention may affect the invasion and persistence of *Wolbachia* in release sites where larval habitats are scarce and/or intermittent.

**IMPORTANCE:**

*Wolbachia* mosquito releases are expanding around the world with substantial impacts on dengue transmission. Releases have succeeded in many locations, but the establishment of *Wolbachia* has been challenging in some environments, and the factors contributing to this outcome remain unresolved. Here, we explore the effects of *Wolbachia* on a novel trait, egg retention, which is likely to be important for the persistence of mosquito populations in locations with intermittent rainfall. We find substantial impacts of the *Wolbachia* strain *w*MelM on the quality of retained eggs but not the *w*AlbB strain. This cost is driven by the *Wolbachia* infection status of the female and can partially recover following a second blood meal. The results of our study may help to explain the difficulty in establishing *Wolbachia* strains at some field release sites and emphasize the need to characterize *Wolbachia* phenotypes across a variety of traits and strains.

## INTRODUCTION

*Aedes aegypti*, commonly known as the yellow fever mosquito, is widespread in tropical regions with high rainfall (1) but also possesses several adaptations that allow them to tolerate dry climates. Their eggs are desiccation tolerant and can remain viable in a quiescent state for several months (2). Moreover, at least under laboratory conditions, females can retain viable eggs in their bodies when oviposition sites are unavailable. Eggs of *Ae. aegypti* and those of at least several other mosquito species can be retained for weeks at a time with little decline in viability (3–5). This includes domestic forms of *Ae. aegypti* that live in close proximity to humans and have adapted to lay eggs in human-made containers (6). These females typically mate once and store sperm in spermathecae to produce offspring for their entire life (7). Mating and blood feeding can occur in either order prior to oviposition (8), and females that have blood fed can retain eggs in their ovaries until finding a suitable oviposition site (9). This provides *Ae. aegypti* with the flexibility to pause their reproduction for extended periods. This ability to retain eggs can be affected by genetic factors (10), but it is unclear whether this variation is driven by ecological differences in conditions experienced by populations. Egg retention could also be affected by other inherited factors, including maternally inherited endosymbionts, such as *Wolbachia*, residing naturally or increasingly being introduced deliberately into mosquitoes for disease control (11). Egg dormancy effects have previously been documented for natural *Wolbachia* in *Drosophila melanogaster* (12). Any effects of *Wolbachia* endosymbionts deliberately introduced to control arbovirus transmission could influence the success of programs aimed at using endosymbionts for disease control. *Wolbachia* from *Drosophila* and other mosquito species have been transferred to *Ae. aegypti* (which do not harbor *Wolbachia* naturally) and are now being released for arbovirus control around the world (13). Several strains of *Wolbachia* block the transmission of arboviruses, including dengue (14, 15), and can induce cytoplasmic incompatibility, which reduces the viability of eggs produced by females that do not carry *Wolbachia* when they mate with males that do (14, 16). Field trials deploying mosquitoes with the *w*Mel *Wolbachia* strain in Australia have established *Wolbachia*-carrying mosquitoes at stable high frequencies in the population (17) and subsequently nearly eliminating local dengue transmission (18, 19). Later releases in dengue-endemic regions involving the *w*Mel or *w*AlbB strains of *Wolbachia* have reduced dengue cases by over 60% in some trial zones in Indonesia (20), Malaysia (21), Colombia (22), and Brazil (23).

Population replacement programs of this nature, where *Wolbachia* are introduced into populations, involve the spread and persistence of *Wolbachia* in wild *Ae. aegypti* populations, which in turn depends on the fidelity of maternal transmission, strength of cytoplasmic incompatibility, and host fitness costs induced by *Wolbachia* (24). Stable establishment of *Wolbachia* has been challenging in some environments (25, 26), which is likely driven by a complex set of factors, including characteristics of the released mosquito strain like pesticide resistance (27), environmental effects on cytoplasmic incompatibility and maternal transmission (28), as well as aspects of the built environment including dispersal barriers and heterogeneity in larval habitats (29, 30). Understanding which factors contribute to *Wolbachia* establishment could help guide decisions about where and when to release, the choice of *Wolbachia* strain, mass rearing procedures, and which locations may require supplementary releases or boosted numbers.

**Fig 1:**
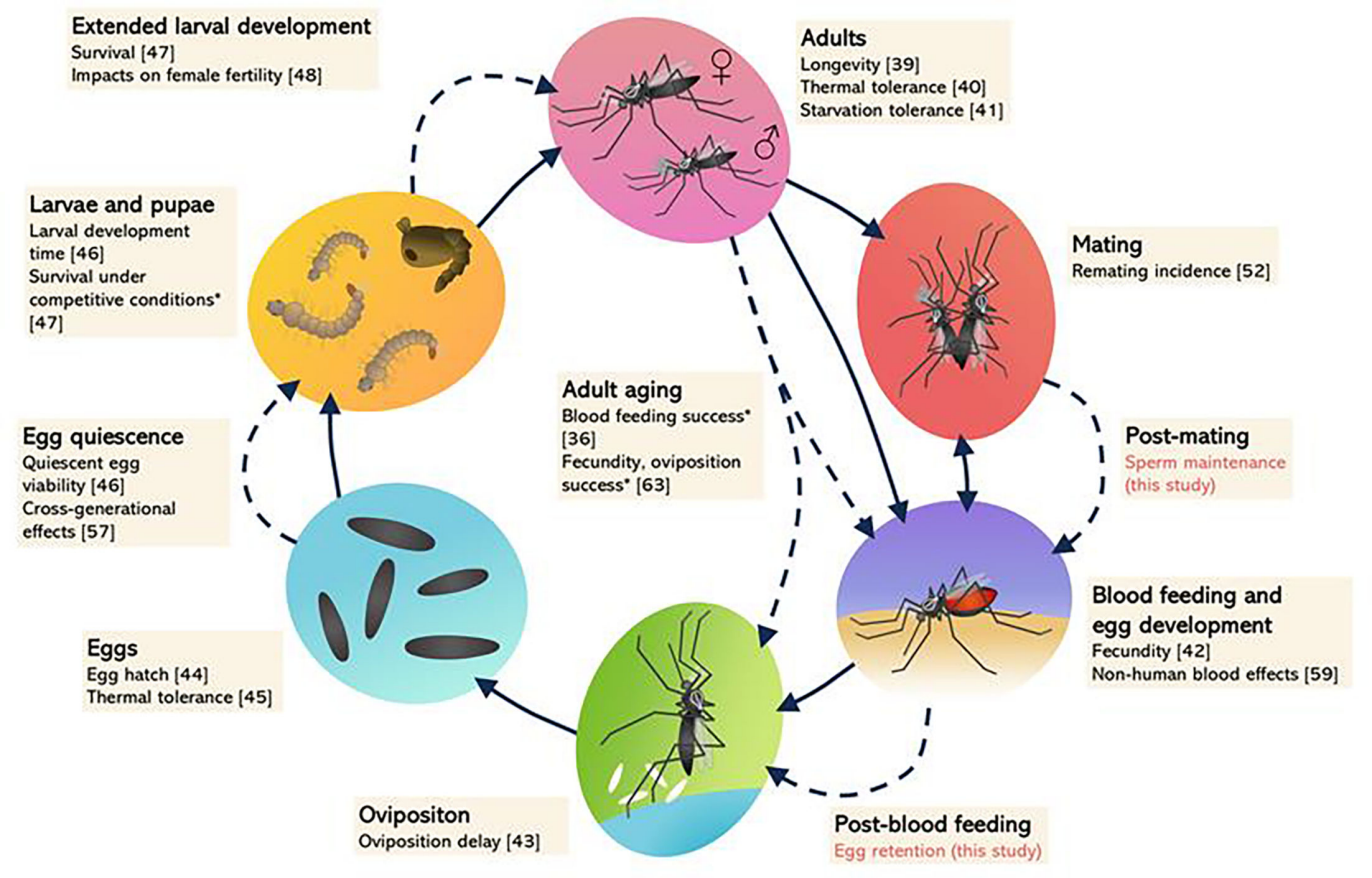
The life cycle of *Ae. aegypti* mosquitoes with examples of host fitness costs induced by *Wolbachia* strains for a range of traits. Solid lines indicate progression across life stages, while dashed lines indicate an extension or pause at a particular stage such as adult aging and egg quiescence. Traits with asterisks (*) are only affected by the highly virulent *w*MelPop infection, which is not currently being released in field trials. Note that costs are strain dependent and may not be identified consistently across studies. Traits that have been tested and found to not be influenced by *Wolbachia* strains such as mating success are not included here except when tested in this study. See references (39–48).

There is now a substantial body of evidence for host fitness costs of *Wolbachia* strains across the *Ae. aegypti* life cycle from egg to adult [reviewed in reference (13), Fig. 1]. Many of these costs are both life-stage-specific and context-dependent, occurring under conditions of environmental stress and amplified during quiescence and senescence. Costs strongly depend on the *Wolbachia* strain (14, 31) and are influenced by genetic background (32, 33). There may also be interactions between the two factors, with studies in different mosquito backgrounds identifying contrasting patterns of fitness costs with the same strains (34, 35). On the other hand, in *Aedes* mosquitoes, many traits do not appear to be influenced by *Wolbachia,* including mating success (36), host-seeking (37), and insecticide resistance (38).

*Wolbachia* releases are occurring in a range of climates, some of them with low and variable rainfall. Understanding the impacts of *Wolbachia* strains on traits such as egg retention relevant to such conditions could be helpful for predicting *Wolbachia* spread in the field. *w*AlbB and *w*MelM are two *Wolbachia* strains now being released for dengue control in different countries, including arid environments such as Jeddah, Saudi Arabia (49). *w*MelM was originally transferred from *Drosophila* melanogaster and is a variant of the widely released *w*Mel strain, which is relatively more heat resistant and has strong dengue-blocking potential (50, 51). Both the *w*AlbB and *w*MelM strains have impacts on fertility, particularly in quiescent states, and stress tolerance (50, 51). We, therefore, measured the impact of both strains on the quantity and quality of eggs when females were forced to retain them under laboratory conditions. Furthermore, we investigated the potential effects of *w*MelM on the quality of stored sperm given that females mating with males carrying *w*MelM have an increased remating frequency (52) and that other *Wolbachia* strains in *Drosophila simulans* reduce sperm competitive ability (53) and the ability of females to store sperm (L. Ferguson, P. A. Ross, and B. van Heerwaarden, unpublished data). We find substantial impacts of *w*MelM but not *w*AlbB on egg hatch following extended egg retention. This effect of *w*MelM occurred regardless of whether females mated with *w*MelM or uninfected males, and some impacts persisted even when females laid a second batch of eggs without retention. In contrast, *w*MelM had no impact on the quality of stored sperm, with declines in fertility largely driven by female age. While the importance of egg retention in the wild has yet to be quantified, the impact of *Wolbachia* on this trait may influence the spread of *Wolbachia* and help to explain heterogeneity in invasion success in some environments.

## MATERIALS AND METHODS

### Mosquito populations and rearing

We used three *Ae. aegypti* populations in this study on a common (North Queensland, Australia) genetic background. Mosquitoes carrying the *w*MelM variant of *Wolbachia* were generated through microinjection of cytoplasm from field-collected *D. melanogaster* as described previously (50). Mosquitoes carrying the *w*AlbB strain (*w*AlbB-Hou variant) were generated through microinjection of the strain generated by Xi et al. (16) to *Ae. aegypti* with an Australian genetic background (54). Uninfected mosquitoes were generated by curing the *w*MelM population of *Wolbachia* by treating adults with 2 mg mL^−1^ tetracycline hydrochloride in a 10% sucrose solution across two consecutive generations. Mosquito populations have been maintained for several years by regular backcrossing of females from each of the three populations to males from a naturally uninfected laboratory population originating from Cairns, North Queensland to avoid genetic drift between lines. Prior to experiments, females from all three populations were backcrossed to this population (so infected and uninfected lines have a similar genetic background and genetic diversity) for an additional two generations. Mosquitoes for colonies and all experiments were maintained under controlled laboratory conditions at 26°C with a 12:12 light:dark cycle according to methods described previously (55).

To rear mosquitoes for experiments, eggs from colonies (<2 weeks old) were hatched in trays with reverse osmosis (RO) water and a few grains of yeast. First instar larvae were transferred to trays with 4 L of RO water at a density of 400 larvae per tray and provided with Hikari Tropical Sinking Wafers (Kyorin food, Himeji, Japan) *ad libitum*. Pupae were sexed and allowed to emerge into separate cages (19.7-L BugDorm-1, MegaView Science Co.*,* Ltd.*,* Taichung City*,* Taiwan) before establishing crosses (see below) when adults were 2–3 days old. Adults were provided with water and a 10% sucrose solution until 1 day before blood feeding when sugar was removed.

### Egg retention and oviposition

**Fig 2:**
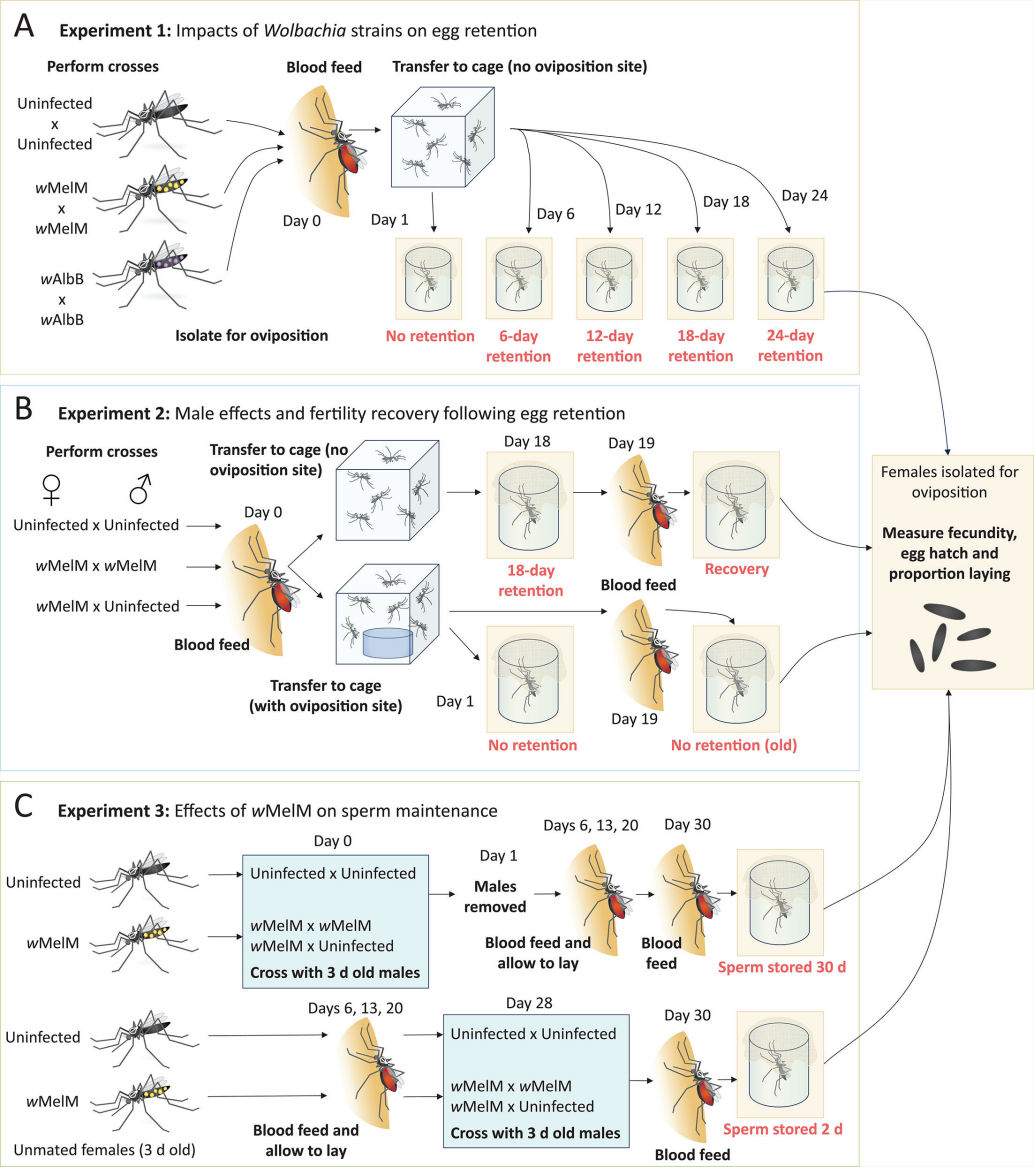
Experimental design. In Experiment 1 (**A**), mated females from the uninfected, wMelM and wAlbB populations experienced no egg retention, or 6, 12, 18, or 24 days of egg retention before laying eggs. In Experiment 2 (**B**), *w*MelM and uninfected females were crossed in different combinations. Females then either experienced no egg retention or 18 days of egg retention. Females from both groups were then blood fed again to initiate a second gonotrophic cycle. In Experiment 3 (**C**), uninfected and *w*MelM females were crossed to 3-day-old males when they were either 3 or 31 days old, then blood fed and isolated for oviposition either 30 or 2 days after mating. Text in red indicates the treatments measured in each experiment for each *Wolbachia* strain or cross. All treatments were measured for fecundity, egg hatch proportion, and the proportion of females laying eggs. For Experiments 1 and 2, Day 0 corresponds to the day of first blood feeding, while for Experiment 3, Day 0 corresponds to when mosquitoes were first mated.

Methods for forcing egg retention were adapted from a previous study (10). Female mosquitoes (5–6 days old) were blood fed on the forearm of a human volunteer (University of Melbourne human ethics approval 0723847) and transferred to BugDorm-1 cages provided with only sugar through a cotton wick (Fig. 2). Oviposition sites were removed, and any water droplets within the cage were wiped up. Open containers of water were placed on top of each cage and cages were then placed within plastic bags. This procedure largely prevented mosquitoes from laying eggs while maintaining a high humidity to reduce mortality. For each treatment, we set up at least three cages (with approximately 200 females and 200 males each) and only used mosquitoes from cages where no eggs were visible. In some cases, mosquitoes laid a small number of eggs on the sugar wick, and these cages were discarded.

Female mosquitoes from egg retention cages were isolated in 70-mL specimen cups at different time points post-blood feeding (Fig. 2). Cups were filled with 20 mL of larval rearing water, lined with strips of sandpaper (Norton Master Painters P80 sandpaper, Saint-Gobain Abrasives Pty. Ltd., Thomastown, VIC, Australia), and covered with a mesh lid to prevent mosquitoes from escaping. Cups were checked daily for 3 days, and sandpaper strips were collected from females that laid eggs. We recorded the number of females that died as well as the number that did not lay eggs after 3 days. Sandpaper strips were partially dried on a paper towel and then placed in sealed containers with a layer of paper towel for 3 days. Eggs were hatched by filling trays with water and adding a few grains of yeast. The next day, the number of unhatched and hatched eggs (with a clearly detached cap) was counted to determine fecundity and egg hatch for individual females.

### Experiment 1—impacts of *Wolbachia* strains on egg retention

We first performed a pilot experiment to measure the impact of egg retention on fecundity and egg hatch proportions for the uninfected, *w*MelM, and *w*AlbB populations. Males and females were crossed in groups in cages (i.e., mass mated with approximately 200 females and 200 males per cage) within populations only. Females were kept in cages with no access to oviposition sites for 1 (no retention), 12, or 18 days post-blood feeding before isolating them for oviposition. We set up 30 replicate cups for the 1- and 12-day time points and 20 replicates for the 18-day time point. For the main experiment (Fig. 2A), we used a similar design but included additional time points (no retention and 6-, 12-, 18-, and 24-day retention), with 40 replicates per time point and population, except for 24 days where we set up 60 replicates per population. Individuals were sampled evenly from three replicate cages, which were tracked separately to test for the effects of replicate cage in the analysis. Females that did not lay eggs were dissected to check for insemination.

### Experiment 2—male effects and fertility recovery following egg retention

In the second experiment (Fig. 2B), we compared the uninfected and *w*MelM populations again and included an additional cross and treatment. Our aims were to (i) test whether the costs of *w*MelM to egg retention were driven by females or males; and (ii) test the extent of fertility recovery in a subsequent gonotrophic cycle after extended egg retention. Uninfected mosquitoes were crossed in groups within the line, and *w*MelM females were crossed to either *w*MelM males or uninfected males in groups. After blood feeding, half the mosquitoes were transferred to egg retention cages with no oviposition site and then isolated for egg laying at 18 days post-blood feeding. To test for recovery, females that survived after 18 days of egg retention (24–25 days old) were blood fed again to collect their eggs from a second gonotrophic cycle. The other half of the mosquitoes were transferred to colony cages containing an oviposition site. Females from this cage were isolated for oviposition 1 day later as a “no egg retention control,” and the remainder were allowed to lay eggs freely. These mosquitoes were blood fed again at 24–25 days old and isolated to collect eggs from a second gonotrophic cycle. We set up 60 replicates per cross for the two controls (5–6-day-old females with no egg retention and 24–25-day-old females with no egg retention in either gonotrophic cycle) and 120 replicates for the 18-day egg retention crosses. All females that survived the 18-day egg retention treatment were blood fed and isolated again for a second gonotrophic cycle, though initial sample sizes were lower than 120 due to mortality. In this experiment, we recorded the proportion of surviving females that laid eggs; those that died were excluded from the analysis.

### Experiment 3—effects of *w*MelM on the quality of stored sperm

In the third experiment (Fig. 2C), we compared the fertility of females that were either mated when they were 3 or 31 days old and then blood fed at 33 days old. Females in all treatments were blood fed four times, with females isolated following the fourth blood meal to measure fertility. In the long-term sperm maintenance treatment (sperm stored for 30 days), females were given 24 hours to mate with males in groups and then all males were removed to ensure that they could not remate for the next 30 days. In the second treatment (sperm stored for 2 days), unmated females were kept separate from males and then mated to 3-day-old males in groups for 24 hours. In both treatments, uninfected mosquitoes were crossed within the line, and *w*MelM females were crossed to either *w*MelM males or uninfected males. The effects of long-term sperm maintenance were measured by comparing the fecundity and egg hatch of females that were blood fed either 2 or 30 days after mating with males.

### Statistics

All statistical analyses were performed in SPSS Statistics version 29.0.0.0. For all experiments, we used general linear models to test for the effects of *Wolbachia* strain, cross and egg retention, or sperm maintenance treatment and their interactions on fecundity and egg hatch proportions (which were logit transformed for normality [56]). Females that died or laid no eggs were excluded from the analyses of fecundity and egg hatch. Tukey’s *post hoc* with multiple comparisons was used to compare treatments within a cross or *Wolbachia* strain. For Experiment 1, we first performed an analysis to test for the effects of replicate cage (nested within line) as a random factor on fecundity and egg hatch. No significant effects of replicate cage or interactions with replicate cage were found, and this factor was removed from subsequent analyses. For Experiments 2 and 3, we performed an initial analysis with only the *w*MelM female × *w*MelM male and *w*MelM female × uninfected male crosses to test whether male strain had any influence on fecundity and egg hatch following egg retention or long-term sperm maintenance. Given the absence of male effects, we then pooled these two crosses for additional analyses focused on testing the effects of *w*MelM in the females. In Experiment 2, where a substantial proportion of females laid no eggs, we also computed the proportions of mosquitoes laying eggs in each treatment, which we analyzed with Fisher’s exact tests treating each female as a data point. We calculated the total number of viable offspring per female by multiplying the number of eggs laid by their hatch proportion. By also including data from females that failed to lay any eggs, this measure provides an overall indication of the total fitness effects of the infection.

## RESULTS

### Impacts of *Wolbachia* strain on egg retention

To test for the effects of *Wolbachia* strain on egg retention, we first performed a pilot experiment where eggs from uninfected, *w*MelM, and *w*AlbB populations were collected and hatched after females experienced 0, 12, or 18 days of forced egg retention. We found a substantial cost of *w*MelM to the hatch proportions of retained eggs relative to the other two populations (Fig. S1), with a significant interaction between *Wolbachia* strain and egg retention treatment (*F*_4,208_ = 5.747, *P* = 0.002). *w*MelM females had a median egg hatch proportion that was 71.5% lower than the control after 12 days and 74.5% lower after 18 days (Fig. S1). While *w*AlbB females had lower egg hatch proportion overall compared to uninfected females, the percentage of decline in hatch proportion with egg retention was similar (9.4% for uninfected and 12.8% for *w*AlbB after 18 days).

**Fig 3:**
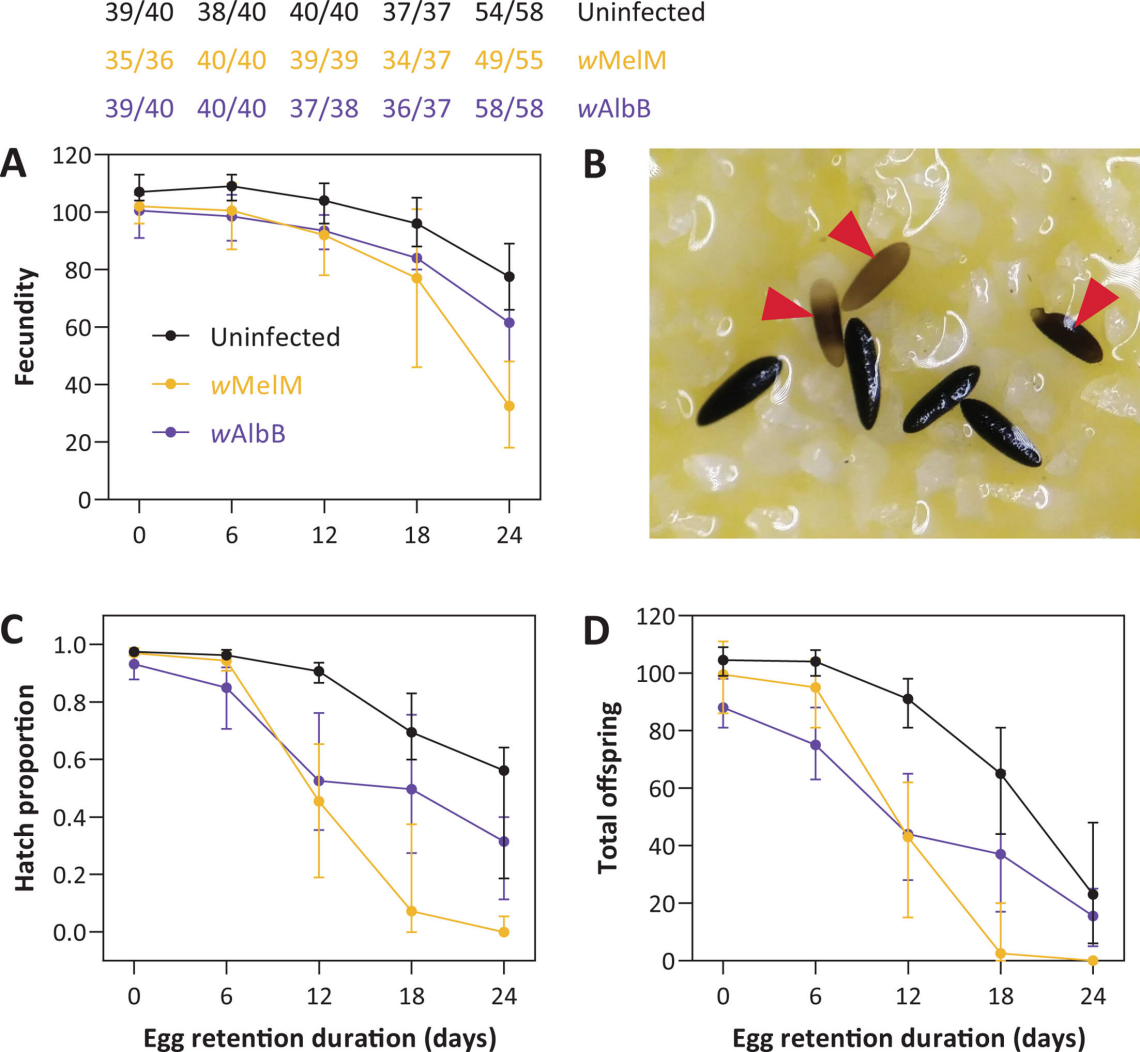
Costs of *Wolbachia* to the quality of retained eggs depend on *Wolbachia* strain. (**A**) Fecundity, (**C**) egg hatch proportions, and (**D**) total offspring produced by uninfected, *w*MelM, or *w*AlbB populations of *Ae. aegypti* after 0, 6, 12, 18, or 24 days of forced retention. Dots show medians and error bars show 95% confidence intervals. Numbers in panel A indicate the number of females in each treatment that laid eggs out of the total number surviving. (**B**) Example of egg defects observed in eggs laid by *w*MelM females after extended egg retention, where eggs with red arrows are unmelanized and eggs with no arrows show typical morphology. An additional example is presented in Fig. S1C.

In the main experiment, where females experienced 0, 6, 12, 18, or 24 days of forced egg retention (Fig. 2A), we found a significant effect of egg retention treatment on both fecundity (GLM: *F*_4,621_ = 52.100, *P* < 0.001) and egg hatch proportion (*F*_4,600_ = 144.580, *P* < 0.001), where both traits in all populations experienced a decline with extended egg retention (Fig. 3). For fecundity, there was a significant effect of *Wolbachia* strain (*F*_2,621_ = 14.276, *P* < 0.001), where *Wolbachia*-infected females had lower fecundity than uninfected females (Fig. 3A), but there was no significant interaction between *Wolbachia* strain and egg retention treatment (*F*_8,621_ = 1.485, *P* = 0.159).

Egg hatch proportions were also influenced by *Wolbachia* strain (*F*_2,208_ = 40.170, *P* < 0.001), and there was a significant interaction between *Wolbachia* strain and egg retention treatment (*F*_8,600_ = 6.770, *P* < 0.001). Consistent with the pilot experiment (Fig. S1), the viability of retained eggs decreased to a much greater extent for *w*MelM females than both *w*AlbB and uninfected females, with close to zero offspring produced by *w*MelM when eggs were retained for 18 or 24 days (Fig. 3D). These results suggest a strain-specific effect of *Wolbachia* on the quality of retained eggs. During this experiment, inspection under a dissecting microscope revealed defects with eggs that were misshapen or unmelanized (Fig. 3B; Fig. S1C provide an example).

### Male effects on egg retention and fertility recovery

We were interested in testing if the costs of *w*MelM to retained egg quality may be mediated by infections in both the male and female and if these could potentially recover in subsequent gonotrophic cycles. We, therefore, tested the impact of *w*MelM on retained egg quality when females were crossed to either *w*MelM or uninfected males. We also tested whether fertility recovered following an additional gonotrophic cycle without egg retention. We first analyzed the data from *w*MelM females that were crossed to *w*MelM or uninfected males. While there was a substantial effect of egg retention treatment on fecundity (GLM: *F*_3,353_ = 31.410, *P* < 0.001) and egg hatch proportion (*F*_3,353_ = 148.878, *P* < 0.001), the male strain used in crosses had no significant effect on either trait (fecundity: *F*_1,353_ = 0.1.026, *P* = 0.749, egg hatch: *F*_1,353_ = 2.924, *P* = 0.088, Fig. S2). However, there was a significant interaction between male strain and egg retention treatment for egg hatch (*F*_3,353_ = 4.921, *P* = 0.002) though not for fecundity (*F*_3,353_ = 0.179, *P* = 0.911).

**Fig 4:**
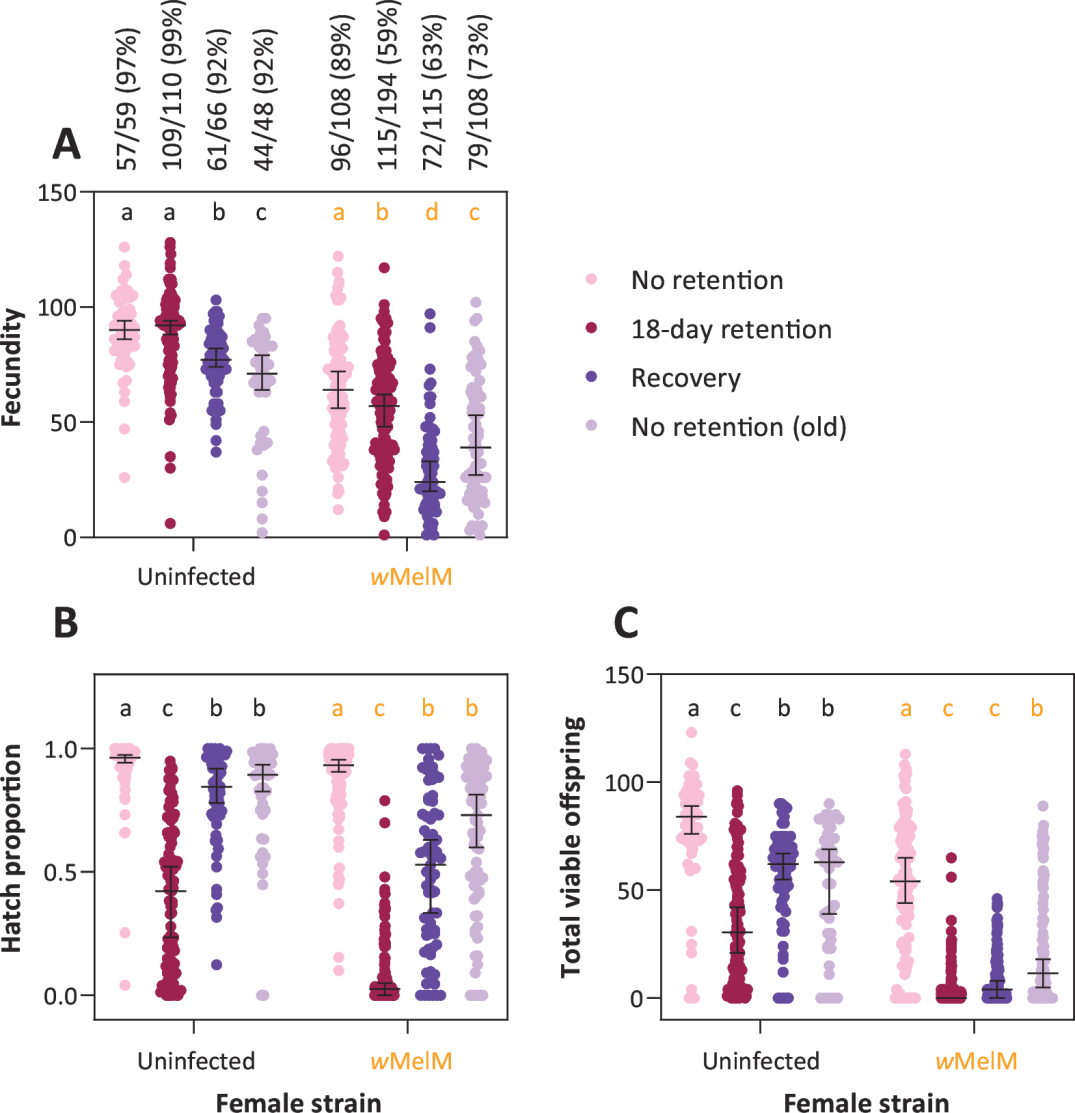
Fertility of *w*MelM females only partially recovers from extended egg retention. (**A**) Fecundity, (**B**) hatch proportions, and (**C**) total viable offspring from uninfected and *w*MelM females. Females were blood fed at 5–6 days old and experienced no retention (pink) or 18 days of egg retention (maroon). Females were also blood fed at 24–25 days old following 18 days of egg retention (dark purple) or no egg retention (light purple) for an additional gonotrophic cycle. For the full experimental design, see Fig. 2B. Data for *w*MelM are pooled from crosses with both uninfected and *w*MelM males (see Fig. S2 for male data). Numbers and percentages in panel A indicate the number and percentage of females in each treatment that laid eggs out of the total number surviving. Dots show data for individual females, while horizontal lines and error bars show medians and 95% confidence intervals. Within each strain, different letters represent significant differences (*P* < 0.05) between egg retention treatments according to Tukey’s *post hoc* tests with a correction for multiple comparisons.

We then pooled data from different male strains to analyze the effects of *Wolbachia* strain on egg retention in females. We found significant effects of female strain (GLM: *F*_1,620_ = 304.010, *P* < 0.001), egg retention treatment (*F*_3,620_ = 42.310, *P* < 0.001), and an interaction between strain and treatment (*F*_3,620_ = 8.027, *P* < 0.001) for fecundity. The fecundity of females laying retained eggs declined for *w*MelM but not for uninfected females compared to females in the control with no egg retention (Fig. 4A). When comparing the two treatments where females were 24–25 days old and in their second gonotrophic cycle, uninfected females laid more eggs when they previously experienced egg retention, while *w*MelM females laid fewer eggs (Fig. 4A).

The proportion of females laying eggs did not differ significantly between *w*MelM and uninfected females in the control (Fisher’s exact test: *P* = 0.142), but for females in the egg retention treatment, significantly fewer *w*MelM females laid eggs (*P* < 0.001, Fig. 4A). There was also an apparent effect of age, with decreased proportions of 24–25-day-old *w*MelM females laying eggs, even for females that experienced no retention (*P* = 0.010, Fig. 4A). There was minimal mortality of isolated females across the experiment, except for the recovery treatment, where only 66/110 (60%) of uninfected and 115/194 (59.3%) of *w*MelM females survived after a second blood meal. We also found significant effects of strain (GLM: *F*_1,620_ = 84.880, *P* < 0.001), treatment (*F*_3,620_ = 182.007, *P* < 0.001), and an interaction (*F*_3,620_ = 11.073, *P* < 0.001) on (logit transformed) egg hatch proportions (Fig. 4B). Egg hatch proportion declined in both strains following extended egg retention, but to a much greater extent for *w*MelM females (median 0.026) than for uninfected females (median 0.422) (Fig. 4B). Egg hatch proportion recovered in the following gonotrophic cycle for both strains, resulting in proportions that were similar to those obtained with females of the same age that experienced no egg retention (Fig. 4B). Egg retention substantially reduced total offspring counts (which included females that laid no eggs), particularly for *w*MelM females where median offspring declined to zero (Fig. 4C). The decline in offspring count with female age was also particularly pronounced for *w*MelM females (Fig. 4C). However, the effects of *w*MelM were not due to age alone since egg hatch proportions, total offspring counts, and the proportion of females laying eggs were lower in the egg retention treatment compared to old females that experienced no egg retention (Fig. 4). These patterns indicate a substantial impact of *w*MelM on fertility with both age and egg retention, with sustained impacts even when completing a second gonotrophic cycle without retention.

### Effects of *w*MelM on long-term sperm maintenance

In the previous experiment, *w*MelM had negative effects on fertility particularly in older females. While this may reflect a deterioration of female fertility with age, costs may also be associated with sperm quality given that females use stored sperm in subsequent gonotrophic cycles. We, therefore, tested for impacts of *w*MelM on long-term sperm maintenance by comparing the fecundity and egg hatch of females that were blood fed either 2 or 30 days after mating. Crosses involving both *w*MelM and uninfected mosquitoes allowed us to separate the effects of *w*MelM in males on stored sperm quality and the effect of *w*MelM in females on long-term sperm maintenance.

When considering only *w*MelM females that mated with either *w*MelM or uninfected males, we found an effect of male strain (GLM: *F*_1,72_ = 8.976, *P* = 0.004), sperm maintenance treatment (*F*_1,72_ = 7.149, *P* = 0.009), and their interaction (*F*_1,72_ = 4.843, *P* = 0.031) on fecundity (Fig. S3). Fecundity was significantly higher in females using sperm stored for 2 days compared to females using sperm stored for 30 days at the point of blood feeding (*F*_1,72_ = 7.149, *P* = 0.009), with this difference being more pronounced when females were crossed to uninfected males (Fig. S3). In contrast, there was no significant effect of any factor or interaction on egg hatch proportion (all *P* > 0.095, Fig. S3).

**Fig 5:**
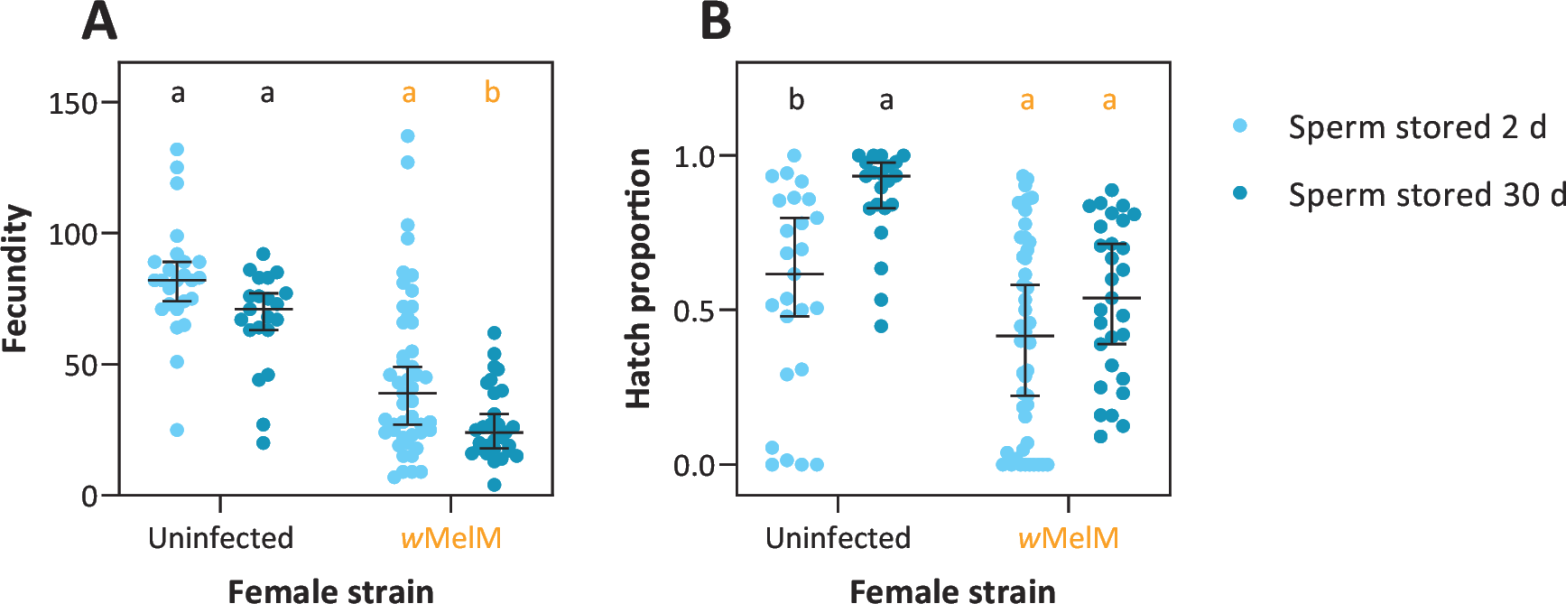
Costs of *w*MelM to fecundity and egg hatch are unaffected by long-term sperm maintenance. (**A**) Fecundity and (**B**) hatch proportions of eggs laid by uninfected or *w*MelM females crossed to 3-day-old males when they were either 3 or 31 days old, then blood fed 2 days (light blue) or 30 days (dark blue) after mating, respectively. For the full experimental design, see Fig. 2C. Dots show data for individual females, while horizontal lines and error bars show medians and 95% confidence intervals. Within each strain, different letters represent significant differences (*P* < 0.05) between sperm maintenance treatments according to GLMs with Tukey’s *post hoc* tests involving a correction for multiple comparisons.

When comparing the effects of female strain with data pooled across *w*MelM and uninfected males, we found that *w*MelM significantly reduced fecundity (*F*_1,118_ = 70.42, *P* < 0.001) and (logit transformed) egg hatch proportion (*F*_1,118_ = 19.15, *P* < 0.001, Fig. 5). Females with sperm stored for 30 days had a lower fecundity compared to females with sperm stored for 2 days (*F*_1,118_ = 13.94, *P* < 0.001), but the hatch proportions of their eggs were higher (*F*_1,118_ = 15.45, *P* < 0.001). Patterns with respect to sperm maintenance treatment were consistent across both wMelM and uninfected females (Fig. 5), with no significant interaction between sperm maintenance treatment and female strain for either fecundity (*F*_1,118_ = 0.092, *P* = 0.762) or egg hatch proportion (*F*_1,118_ = 3.065, *P* = 0.083), suggesting that *w*MelM has no deleterious effect on the quality of stored sperm specifically.

## DISCUSSION

We show that *Wolbachia* strain *w*MelM disrupts the ability of *Ae. aegypti* females to retain viable eggs for extended periods, with a decrease in the proportion of females that lay eggs, as well as a substantial reduction in the proportion of those eggs that hatch. The phenotype described here adds to a growing list of traits influenced by *Wolbachia* strains in *Ae. aegypti* (Fig. 1) and is consistent with other studies that have described context-dependent and strain-dependent fitness costs, particularly to traits related to fertility (57). The costs described here and in other studies could contribute to challenges in establishing this strain in field populations, especially in climates with intermittent rainfall where mosquitoes may frequently retain eggs.

Our comparison of the *w*AlbB and *w*MelM strains shows that the costs of *Wolbachia* are strain-specific since no clear effects of *w*AlbB on fecundity or egg hatch following retention were identified. The lack of effect of *w*AlbB was surprising given that substantial costs of this strain have been identified previously, particularly when mosquito eggs are in quiescent states (e.g., references [57, 58], though other studies do show higher costs of a different *w*Mel variant compared to wAlbB under some conditions [34, 35]). Retained eggs laid by *w*MelM females often had low hatch proportions, which may be partially explained by the presence of defects such as incomplete melanization, which were rarely detected in the uninfected and *w*AlbB populations. These defects are similar to those of eggs laid by females carrying the *w*MelPop *Wolbachia* strain when females are aged or fed on non-human blood (59), but whether a common mechanism is involved remains to be explored.

Under some conditions, such as nutrient deprivation, *Ae. aegypti* females can resorb their oocytes (60, 61). In most experiments, we found a minimal decline in fecundity for uninfected females following extended egg retention. In contrast, *w*MelM females tended to lay fewer eggs under the same conditions, and many females laid no eggs in the second experiment. This may reflect females resorbing their eggs or possibly continued retention of eggs despite access to an oviposition site. Dissections could be used to further explore this issue. Our experiments show that costs of *w*MelM were driven mainly by the female since effects occurred regardless of the male used in the crosses. In one experiment where females that did not lay eggs were dissected to check for insemination, all had visible sperm in two to three spermathecae, suggesting that mating was not an issue. Our sperm maintenance experiments also suggest that *w*MelM has no impact on sperm quality/sperm viability or on female ability to store sperm, indicating that costs of *w*Mel relate to the quality of retained eggs or the ability of females to fertilize eggs, which occurs during oviposition (62).

We acknowledge that some of the effects of *w*MelM described in our study could be due to mosquito age rather than solely due to egg retention effects. While *w*MelM does not appear to affect adult longevity (50), previous studies have identified costs of *Wolbachia* to fertility that increase with age (63), and here, we show the costs for *w*MelM when females of different ages (but with no egg retention) are compared. However, total offspring counts for *w*MelM females were lower in the retention treatment compared to those for older females that experienced no retention, highlighting that effects extend beyond age. The effects of *w*MelM on egg retention also appear to persist to some extent since total offspring counts did not fully recover after feeding again, in contrast to uninfected mosquitoes, which fully recovered.

In summary, our study describes the substantial costs of *w*MelM, a *Wolbachia* strain now being released in dengue control programs, to a trait that is likely to be important in environments with variable rainfall. While the experimental conditions used here might be regarded as relatively extreme, the fact that *Ae. aegypti* are capable of extended egg retention while retaining viability does suggest that it may link to an adaptive physiological trait. Furthermore, while the costs of *Wolbachia* to individual traits can be minor, the cumulative costs across many traits (Fig. 1) can add up to be quite substantial under some conditions (51), which could explain fluctuations and loss of *Wolbachia* in some field release sites (25, 64). While we did not consider other environmental factors such as temperature, these may also interact with the effects on egg retention leading to additional costs. The costs described here and elsewhere also raise important evolutionary questions since successful *Wolbachia* establishment will result in mosquito populations that are less tolerant of dry environments if their ability to retain eggs is disrupted. If *Wolbachia* remains at high frequency, these costs may shift over time as recently demonstrated for quiescent egg viability (33). These environment-specific effects raise issues about the long-term success of the widespread replacement of wild populations with mosquitoes carrying *Wolbachia* strains for dengue reduction under climate change (65). It may be that the suitability of different strains changes under more variable future rainfall patterns, particularly if temperature extremes lead to higher evapotranspiration rates.

## SUPPLEMENTAL MATERIAL

Supplemental figures aem.01491-24-s0001.docx Figures S1 to S3.

ASM does not own the copyrights to Supplemental Material that may be linked to, or accessed through, an article. The authors have granted ASM a non-exclusive, world-wide license to publish the Supplemental Material files. Please contact the corresponding author directly for reuse.

## References

[1] Laporta GZ, Potter AM, Oliveira JFA, Bourke BP, Pecor DB, Linton Y-M. 2023. Global distribution of Aedes aegypti and Aedes albopictus in a climate change scenario of regional rivalry. Insects 14:49. doi:10.3390/insects14010049 36661976 PMC9860750

[2] Sota T, Mogi M. 1992. Interspecific variation in desiccation survival time of Aedes (Stegomyia) mosquito eggs is correlated with habitat and egg size. Oecologia 90:353–358. doi:10.1007/BF00317691 28313521

[3] Johnson BJ, Fonseca DM. 2014. The effects of forced-egg retention on the blood-feeding behavior and reproductive potential of Culex pipiens (Diptera: Culicidae). J Insect Physiol 66:53–58. doi:10.1016/j.jinsphys.2014.05.014 24862157

[4] Xue R-D, Ali A, Barnard DR. 2005. Effects of forced egg-retention in Aedes albopictus on adult survival and reproduction following application of DEET as an oviposition deterrent. J Vector Ecol 30:45–48. 16007955

[5] Judson CL. 1968. Physiology of feeding and oviposition behavior in Aedes aegypti (L.): experimental dissociation of feeding and oogenesis. J Med Entomol 5:21–23. doi:10.1093/jmedent/5.1.21 5642171

[6] Kolimenakis A, Heinz S, Wilson ML, Winkler V, Yakob L, Michaelakis A, Papachristos D, Richardson C, Horstick O. 2021. The role of urbanisation in the spread of Aedes mosquitoes and the diseases they transmit-a systematic review. PLoS Negl Trop Dis 15:e0009631. doi:10.1371/journal.pntd.0009631 34499653 PMC8428665

[7] Degner EC, Harrington LC. 2016. Polyandry depends on postmating time interval in the dengue vector Aedes aegypti*.* Am J Trop Med Hyg 94:780–785. doi:10.4269/ajtmh.15-0893 26880776 PMC4824218

[8] League GP, Degner EC, Pitcher SA, Hafezi Y, Tennant E, Cruz PC, Krishnan RS, Garcia Castillo SS, Alfonso-Parra C, Avila FW, Wolfner MF, Harrington LC. 2021. The impact of mating and sugar feeding on blood-feeding physiology and behavior in the arbovirus vector mosquito Aedes aegypti. PLoS Negl Trop Dis 15:e0009815. doi:10.1371/journal.pntd.0009815 34591860 PMC8509887

[9] Bentley MD, Day JF. 1989. Chemical ecology and behavioral aspects of mosquito oviposition. Annu Rev Entomol 34:401–421. doi:10.1146/annurev.en.34.010189.002153 2564759

[10] Venkataraman K, Shai N, Lakhiani P, Zylka S, Zhao J, Herre M, Zeng J, Neal LA, Molina H, Zhao L, Vosshall LB. 2023. Two novel, tightly linked, and rapidly evolving genes underlie Aedes aegypti mosquito reproductive resilience during drought. Elife 12:e80489. doi:10.7554/eLife.80489 36744865 PMC10076016

[11] Ant TH, Mancini MV, McNamara CJ, Rainey SM, Sinkins SP. 2023. Wolbachia-virus interactions and arbovirus control through population replacement in mosquitoes. Pathog Glob Health 117:245–258. doi:10.1080/20477724.2022.2117939 36205550 PMC10081064

[12] Kriesner P, Conner WR, Weeks AR, Turelli M, Hoffmann AA. 2016. Persistence of a Wolbachia infection frequency cline in Drosophila melanogaster and the possible role of reproductive dormancy. Evolution 70:979–997. doi:10.1111/evo.12923 27076356 PMC4874875

[13] Ross PA, Turelli M, Hoffmann AA. 2019. Evolutionary ecology of Wolbachia releases for disease control. Annu Rev Genet 53:93–116. doi:10.1146/annurev-genet-112618-043609 31505135 PMC6944334

[14] Walker T, Johnson PH, Moreira LA, Iturbe-Ormaetxe I, Frentiu FD, McMeniman CJ, Leong YS, Dong Y, Axford J, Kriesner P, Lloyd AL, Ritchie SA, O’Neill SL, Hoffmann AA. 2011. The wMel Wolbachia strain blocks dengue and invades caged Aedes aegypti populations. Nature New Biol 476:450–453. doi:10.1038/nature10355 21866159

[15] Bian G, Xu Y, Lu P, Xie Y, Xi Z. 2010. The endosymbiotic bacterium Wolbachia induces resistance to dengue virus in Aedes aegypti. PLoS Pathog 6:e1000833. doi:10.1371/journal.ppat.1000833 20368968 PMC2848556

[16] Xi Z, Khoo CCH, Dobson SL. 2005. Wolbachia establishment and invasion in an Aedes aegypti laboratory population. Science 310:326–328. doi:10.1126/science.1117607 16224027

[17] Hoffmann AA, Montgomery BL, Popovici J, Iturbe-Ormaetxe I, Johnson PH, Muzzi F, Greenfield M, Durkan M, Leong YS, Dong Y, Cook H, Axford J, Callahan AG, Kenny N, Omodei C, McGraw EA, Ryan PA, Ritchie SA, Turelli M, O’Neill SL. 2011. Successful establishment of Wolbachia in Aedes populations to suppress dengue transmission. Nature 476:454–457. doi:10.1038/nature10356 21866160

[18] O’Neill SL, Ryan PA, Turley AP, Wilson G, Retzki K, Iturbe-Ormaetxe I, Dong Y, Kenny N, Paton CJ, Ritchie SA, Brown-Kenyon J, Stanford D, Wittmeier N, Anders KL, Simmons CP. 2018. Scaled deployment of Wolbachia to protect the community from dengue and other Aedes transmitted arboviruses. Gates Open Res 2:36. doi:10.12688/gatesopenres.12844.2 30596205 PMC6305154

[19] Ryan PA, Turley AP, Wilson G, Hurst TP, Retzki K, Brown-Kenyon J, Hodgson L, Kenny N, Cook H, Montgomery BL, Paton CJ, Ritchie SA, Hoffmann AA, Jewell NP, Tanamas SK, Anders KL, Simmons CP, O’Neill SL. 2019. Establishment of wMel Wolbachia in Aedes aegypti mosquitoes and reduction of local dengue transmission in Cairns and surrounding locations in northern Queensland, Australia. Gates Open Res 3:1547. doi:10.12688/gatesopenres.13061.2 31667465 PMC6801363

[20] Utarini A, Indriani C, Ahmad RA, Tantowijoyo W, Arguni E, Ansari MR, Supriyati E, Wardana DS, Meitika Y, Ernesia I, Nurhayati I, Prabowo E, Andari B, Green BR, Hodgson L, Cutcher Z, Rancès E, Ryan PA, O’Neill SL, Dufault SM, Tanamas SK, Jewell NP, Anders KL, Simmons CP, AWED Study Group. 2021. Efficacy of Wolbachia-infected mosquito deployments for the control of dengue. N Engl J Med 384:2177–2186. doi:10.1056/NEJMoa2030243 34107180 PMC8103655

[21] Hoffmann Ary A., Ahmad NW, Keong WM, Ling CY, Ahmad NA, Golding N, Tierney N, Jelip J, Putit PW, Mokhtar N, Sandhu SS, Ming LS, Khairuddin K, Denim K, Rosli NM, Shahar H, Omar T, Ridhuan Ghazali MK, Aqmar Mohd Zabari NZ, Abdul Karim MA, Saidin MI, Mohd Nasir MN, Aris T, Sinkins SP. 2024. Introduction of Aedes aegypti mosquitoes carrying wAlbB Wolbachia sharply decreases dengue incidence in disease hotspots. i Sci 27:108942. doi:10.1016/j.isci.2024.108942 PMC10847733 38327789

[22] Velez ID, Tanamas SK, Arbelaez MP, Kutcher SC, Duque SL, Uribe A, Zuluaga L, Martínez L, Patiño AC, Barajas J, Muñoz E, Mejia Torres MC, Uribe S, Porras S, Almanza R, Pulido H, O’Neill SL, Santacruz-Sanmartin E, Gonzalez S, Ryan PA, Denton JA, Jewell NP, Dufault SM, Simmons CP, Anders KL. 2023. Reduced dengue incidence following city-wide wMel Wolbachia mosquito releases throughout three Colombian cities: interrupted time series analysis and a prospective case-control study. PLoS Negl Trop Dis 17:e0011713. doi:10.1371/journal.pntd.0011713 38032857 PMC10688673

[23] Pinto SB, Riback TIS, Sylvestre G, Costa G, Peixoto J, Dias FBS, Tanamas SK, Simmons CP, Dufault SM, Ryan PA, O’Neill SL, Muzzi FC, Kutcher S, Montgomery J, Green BR, Smithyman R, Eppinghaus A, Saraceni V, Durovni B, Anders KL, Moreira LA. 2021. Effectiveness of Wolbachia-infected mosquito deployments in reducing the incidence of dengue and other Aedes-borne diseases in Niterói, Brazil: a quasi-experimental study. PLoS Negl Trop Dis 15:e0009556. doi:10.1371/journal.pntd.0009556 34252106 PMC8297942

[24] Hoffmann AA, Turelli MJ. 1997. Cytoplasmic incompatibility in insects, p 42–80. In Influential passengers: inherited microorganisms and arthropod reproduction

[25] Hien NT, Anh DD, Le NH, Yen NT, Phong TV, Nam VS, Duong TN, Nguyen NB, Huong DTT, Hung LQ, et al.. 2021. Environmental factors influence the local establishment of Wolbachia in Aedes aegypti mosquitoes in two small communities in central Vietnam. Gates Open Res 5:147. doi:10.12688/gatesopenres.13347.2 35602266 PMC9098883

[26] Gesto JSM, Pinto SB, Dias FBS, Peixoto J, Costa G, Kutcher S, Montgomery J, Green BR, Anders KL, Ryan PA, Simmons CP, O’Neill SL, Moreira LA. 2021 Large-scale deployment and establishment of Wolbachia into the Aedes aegypti population in Rio de Janeiro, Brazil. Front Microbiol 12:2021. doi:10.3389/fmicb.2021.711107 PMC8356046 34394061

[27] Garcia G de A, Sylvestre G, Aguiar R, da Costa GB, Martins AJ, Lima JBP, Petersen MT, Lourenço-de-Oliveira R, Shadbolt MF, Rašić G, Hoffmann AA, Villela DAM, Dias FBS, Dong Y, O’Neill SL, Moreira LA, Maciel-de-Freitas R. 2019. Matching the genetics of released and local Aedes aegypti populations is critical to assure Wolbachia invasion. PLoS Negl Trop Dis 13:e0007023. doi:10.1371/journal.pntd.0007023 30620733 PMC6338382

[28] Ross PA, Wiwatanaratanabutr I, Axford JK, White VL, Endersby-Harshman NM, Hoffmann AA. 2017. Wolbachia infections in Aedes aegypti differ markedly in their response to cyclical heat stress. PLoS Pathog 13:e1006006. doi:10.1371/journal.ppat.1006006 28056065 PMC5215852

[29] Schmidt TL, Filipović I, Hoffmann AA, Rašić G. 2018. Fine-scale landscape genomics helps explain the slow spatial spread of Wolbachia through the Aedes aegypti population in Cairns, Australia. Heredity (Edinb) 120:386–395. doi:10.1038/s41437-017-0039-9 29358725 PMC5889405

[30] Hancock PA, Ritchie SA, Koenraadt CJM, Scott TW, Hoffmann AA, Godfray HCJ. 2019. Predicting the spatial dynamics of Wolbachia infections in Aedes aegypti arbovirus vector populations in heterogeneous landscapes. J Appl Ecol 56:1674–1686. doi:10.1111/1365-2664.13423

[31] Fraser JE, De Bruyne JT, Iturbe-Ormaetxe I, Stepnell J, Burns RL, Flores HA, O’Neill SL. 2017. Novel Wolbachia-transinfected Aedes aegypti mosquitoes possess diverse fitness and vector competence phenotypes. PLoS Pathog 13:e1006751. doi:10.1371/journal.ppat.1006751 29216317 PMC5736235

[32] Carvalho DO, Torres‐Monzon JA, Koskinioti P, Dilrukshi Wijegunawardana NDA, Liang X, Pillwax G, Xi Z, Bourtzis K. 2020. Aedes aegypti lines for combined sterile insect technique and incompatible insect technique applications: the importance of host genomic background. Entomol Exp Applic 168:560–572. doi:10.1111/eea.12892

[33] Ross PA, Hoffmann AA. 2022. Fitness costs of Wolbachia shift in locally-adapted Aedes aegypti mosquitoes. Environ Microbiol 24:5749–5759. doi:10.1111/1462-2920.16235 36200325 PMC10947380

[34] Joubert DA, Walker T, Carrington LB, De Bruyne JT, Kien DHT, Hoang NLT, Chau NVV, Iturbe-Ormaetxe I, Simmons CP, O’Neill SL. 2016. Establishment of a Wolbachia superinfection in Aedes aegypti mosquitoes as a potential approach for future resistance management. PLoS Pathog 12:e1005434. doi:10.1371/journal.ppat.1005434 26891349 PMC4758728

[35] Maciel-de-Freitas R, Sauer FG, Kliemke K, Garcia GA, Pavan MG, David MR, Schmidt-Chanasit J, Hoffmann A, Lühken R. 2024. Wolbachia strains wMel and wAlbB differentially affect Aedes aegypti traits related to fecundity. Microbiol Spectr 12:e00128-24. doi:10.1128/spectrum.00128-24 38483475 PMC10986601

[36] Turley AP, Zalucki MP, O’Neill SL, McGraw EA. 2013. Transinfected Wolbachia have minimal effects on male reproductive success in Aedes aegypti. Parasites Vectors 6:36. doi:10.1186/1756-3305-6-36 23399027 PMC3584945

[37] Lau MJ, Endersby-Harshman NM, Axford JK, Ritchie SA, Hoffmann AA, Ross PA. 2020. Measuring the host-seeking ability of Aedes aegypti destined for field release. Am J Trop Med Hyg 102:223–231. doi:10.4269/ajtmh.19-0510 31769394 PMC6947783

[38] Endersby NM, Hoffmann AA. 2013. Effect of Wolbachia on insecticide susceptibility in lines of Aedes aegypti. Bull Entomol Res 103:269–277. doi:10.1017/S0007485312000673 23149015

[39] McMeniman CJ, Lane RV, Cass BN, Fong AWC, Sidhu M, Wang Y-F, O’Neill SL. 2009. Stable introduction of a life-shortening Wolbachia infection into the mosquito Aedes aegypti*.* Science 323:141–144. doi:10.1126/science.1165326 19119237

[40] Ware-Gilmore F, Sgrò CM, Xi Z, Dutra HLC, Jones MJ, Shea K, Hall MD, Thomas MB, McGraw EA. 2021. Microbes increase thermal sensitivity in the mosquito Aedes aegypti, with the potential to change disease distributions. PLoS Negl Trop Dis 15:e0009548. doi:10.1371/journal.pntd.0009548 34292940 PMC8297775

[41] Foo I-H, Hoffmann AA, Ross PA. 2019. Cross-generational effects of heat stress on fitness and Wolbachia density in Aedes aegypti mosquitoes. Trop Med Infect Dis 4:13. doi:10.3390/tropicalmed4010013 30642130 PMC6473245

[42] Hoffmann AA, Iturbe-Ormaetxe I, Callahan AG, Phillips BL, Billington K, Axford JK, Montgomery B, Turley AP, O’Neill SL. 2014. Stability of the wMel Wolbachia infection following invasion into Aedes aegypti populations. PLoS Negl Trop Dis 8:e3115. doi:10.1371/journal.pntd.0003115 25211492 PMC4161343

[43] Pimenta de Oliveira S, Dantas de Oliveira C, Viana Sant’Anna MR, Carneiro Dutra HL, Caragata EP, Moreira LA. 2017. Wolbachia infection in Aedes aegypti mosquitoes alters blood meal excretion and delays oviposition without affecting trypsin activity. Insect Biochem Mol Biol 87:65–74. doi:10.1016/j.ibmb.2017.06.010 28655666

[44] Dutra HLC, Dos Santos LMB, Caragata EP, Silva JBL, Villela DAM, Maciel-de-Freitas R, Moreira LA. 2015. From lab to field: the influence of urban landscapes on the invasive potential of Wolbachia in Brazilian Aedes aegypti mosquitoes. PLoS Negl Trop Dis 9:e0003689. doi:10.1371/journal.pntd.0003689 25905888 PMC4408005

[45] Ross PA, Ritchie SA, Axford JK, Hoffmann AA. 2019. Loss of cytoplasmic incompatibility in Wolbachia-infected Aedes aegypti under field conditions. PLoS Negl Trop Dis 13:e0007357. doi:10.1371/journal.pntd.0007357 31002720 PMC6493766

[46] Yeap HL, Mee P, Walker T, Weeks AR, O’Neill SL, Johnson P, Ritchie SA, Richardson KM, Doig C, Endersby NM, Hoffmann AA. 2011. Dynamics of the “popcorn” Wolbachia infection in outbred Aedes aegypti informs prospects for mosquito vector control. Genetics 187:583–595. doi:10.1534/genetics.110.122390 21135075 PMC3030498

[47] Ross PA, Endersby NM, Hoffmann AA. 2016. Costs of three Wolbachia infections on the survival of Aedes aegypti larvae under starvation conditions. PLoS Negl Trop Dis 10:e0004320. doi:10.1371/journal.pntd.0004320 26745630 PMC4706305

[48] Lau M-J, Ross PA, Endersby-Harshman NM, Yang Q, Hoffmann AA. 2022. Wolbachia inhibits ovarian formation and increases blood feeding rate in female Aedes aegypti. PLoS Negl Trop Dis 16:e0010913. doi:10.1371/journal.pntd.0010913 36367854 PMC9683608

[49] Endersby-Harshman NM, Ali A, Alhumrani B, Alkuriji MA, Al-Fageeh MB, Al-Malik A, Alsuabeyl MS, Elfekih S, Hoffmann AA. 2021. Voltage-sensitive sodium channel (Vssc) mutations associated with pyrethroid insecticide resistance in Aedes aegypti (L.) from two districts of Jeddah, Kingdom of Saudi Arabia: baseline information for a Wolbachia release program. Parasites Vectors 14:1–13. doi:10.1186/s13071-021-04867-3 34247634 PMC8273952

[50] Gu X, Ross PA, Rodriguez-Andres J, Robinson KL, Yang Q, Lau M-J, Hoffmann AA. 2022. A wMel Wolbachia variant in Aedes aegypti from field-collected Drosophila melanogaster with increased phenotypic stability under heat stress. Environ Microbiol 24:2119–2135. doi:10.1111/1462-2920.15966 35319146 PMC9544352

[51] Ross PA, Elfekih S, Collier S, Klein MJ, Lee SS, Dunn M, Jackson S, Zhang Y, Axford JK, Gu X, Home JL, Nassar MS, Paradkar PN, Tawfik EA, Jiggins FM, Almalik AM, Al-Fageeh MB, Hoffmann AA. 2023. Developing Wolbachia-based disease interventions for an extreme environment. PLoS Pathog 19:e1011117. doi:10.1371/journal.ppat.1011117 36719928 PMC9917306

[52] Osorio J, Villa-Arias S, Camargo C, Ramírez-Sánchez LF, Barrientos LM, Bedoya C, Rúa-Uribe G, Dorus S, Alfonso-Parra C, Avila FW. 2023. wMel Wolbachia alters female post-mating behaviors and physiology in the dengue vector mosquito Aedes aegypti*.* Commun Biol 6:865. doi:10.1038/s42003-023-05180-8 37604924 PMC10442437

[53] Champion de Crespigny FE, Wedell N. 2006. Wolbachia infection reduces sperm competitive ability in an insect. Proc Biol Sci 273:1455–1458. doi:10.1098/rspb.2006.3478 16777737 PMC1560322

[54] Ross PA, Gu X, Robinson KL, Yang Q, Cottingham E, Zhang Y, Yeap HL, Xu X, Endersby-Harshman NM, Hoffmann AA. 2021. A wAlbB Wolbachia transinfection displays stable phenotypic effects across divergent Aedes aegypti mosquito backgrounds. Appl Environ Microbiol 87:e0126421. doi:10.1128/AEM.01264-21 34379518 PMC8478461

[55] Ross PA, Axford JK, Richardson KM, Endersby-Harshman NM, Hoffmann AA. 2017. Maintaining Aedes aegypti mosquitoes infected with Wolbachia. J Vis Exp 2017:56124. doi:10.3791/56124 PMC5614331 28829414

[56] Warton DI, Hui FKC. 2011. The arcsine is asinine: the analysis of proportions in ecology. Ecology 92:3–10. doi:10.1890/10-0340.1 21560670

[57] Lau M-J, Ross PA, Hoffmann AA. 2021. Infertility and fecundity loss of Wolbachia-infected Aedes aegypti hatched from quiescent eggs is expected to alter invasion dynamics. PLoS Negl Trop Dis 15:e0009179. doi:10.1371/journal.pntd.0009179 33591971 PMC7909672

[58] Axford JK, Ross PA, Yeap HL, Callahan AG, Hoffmann AA. 2016. Fitness of wAlbB Wolbachia infection in Aedes aegypti: parameter estimates in an outcrossed background and potential for population invasion. Am J Trop Med Hyg 94:507–516. doi:10.4269/ajtmh.15-0608 26711515 PMC4775882

[59] McMeniman CJ, Hughes GL, O’Neill SL. 2011. A Wolbachia symbiont in Aedes aegypti disrupts mosquito egg development to a greater extent when mosquitoes feed on nonhuman versus human blood. jnl med entom 48:76–84. doi:10.1603/ME09188 21337952

[60] Lea AO, Briegel H, Lea HM. 1978. Arrest, resorption, or maturation of oöcytes in Aedes aegypti: dependence on the quantity of blood and the interval between blood meals. Physiol Entomol 3:309–316. doi:10.1111/j.1365-3032.1978.tb00164.x

[61] Clifton ME, Noriega FG. 2012. The fate of follicles after a blood meal is dependent on previtellogenic nutrition and juvenile hormone in Aedes aegypti*.* J Insect Physiol 58:1007–1019. doi:10.1016/j.jinsphys.2012.05.005 22626792 PMC3389259

[62] Degner EC, Harrington LC. 2016. A mosquito sperm’s journey from male ejaculate to egg: mechanisms, molecules, and methods for exploration. Mol Reprod Dev 83:897–911. doi:10.1002/mrd.22653 27147424 PMC5086422

[63] McMeniman CJ, O’Neill SL. 2010. A virulent Wolbachia infection decreases the viability of the dengue vector Aedes aegypti during periods of embryonic quiescence. PLoS Negl Trop Dis 4:e748. doi:10.1371/journal.pntd.0000748 20644622 PMC2903475

[64] Nazni WA, Hoffmann AA, NoorAfizah A, Cheong YL, Mancini MV, Golding N, Kamarul GMR, Arif MAK, Thohir H, NurSyamimi H, et al.. 2019. Establishment of Wolbachia strain wAlbB in Malaysian populations of Aedes aegypti for dengue control. Curr Biol 29:4241–4248. doi:10.1016/j.cub.2019.11.007 31761702 PMC6926472

[65] Ross PA, Hoffmann AA. 2023. Limits to modeling the (thermal) limits of Wolbachia. EcoEvoRxiv. doi:10.32942/X2VW23

