# Supplementary material for: Wolbachia strain wMelM disrupts egg retention by Aedes aegypti females prevented from ovipositing
Source: Appl Environ Microbiol. 2024 Dec 4;91(1):e01491-24. doi: 10.1128/aem.01491-24 (PMC11784415; doi:10.1128/aem.01491-24)
Supplement: Supplemental figures — Figures S1 to S3. [file aem.01491-24-s0001.docx]

Supplementary information

**
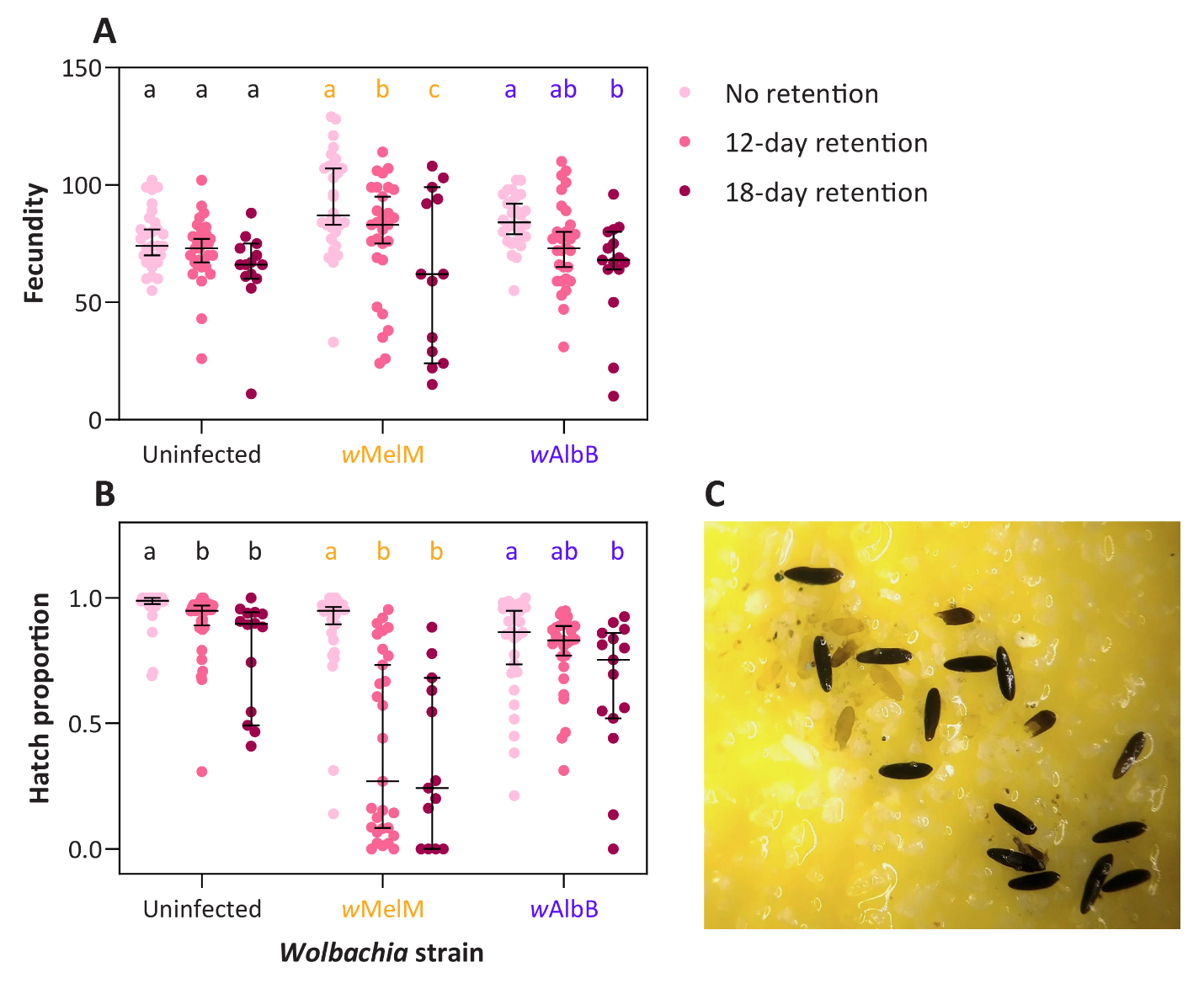
**

**Figure S1. Pilot experiments showing *Wolbachia*-dependent costs to the quality of retained eggs.** (A) Fecundity and (B) hatch proportions of eggs laid by uninfected, *w*MelM or *w*AlbB populations of *Ae. aegypti* after 0, 12 or 18 days of forced retention. Dots show data for individual females while horizontal lines and error bars show medians and 95% confidence intervals. Within each population, different letters represent significant differences (P < 0.05) between egg retention treatments based on Tukey’s post-hoc tests with a correction for multiple comparisons. (C) Example of egg defects observed in eggs laid by *w*MelM females after extended egg retention. Note the mis-formed and unmelanized eggs laid on the oviposition substrate.


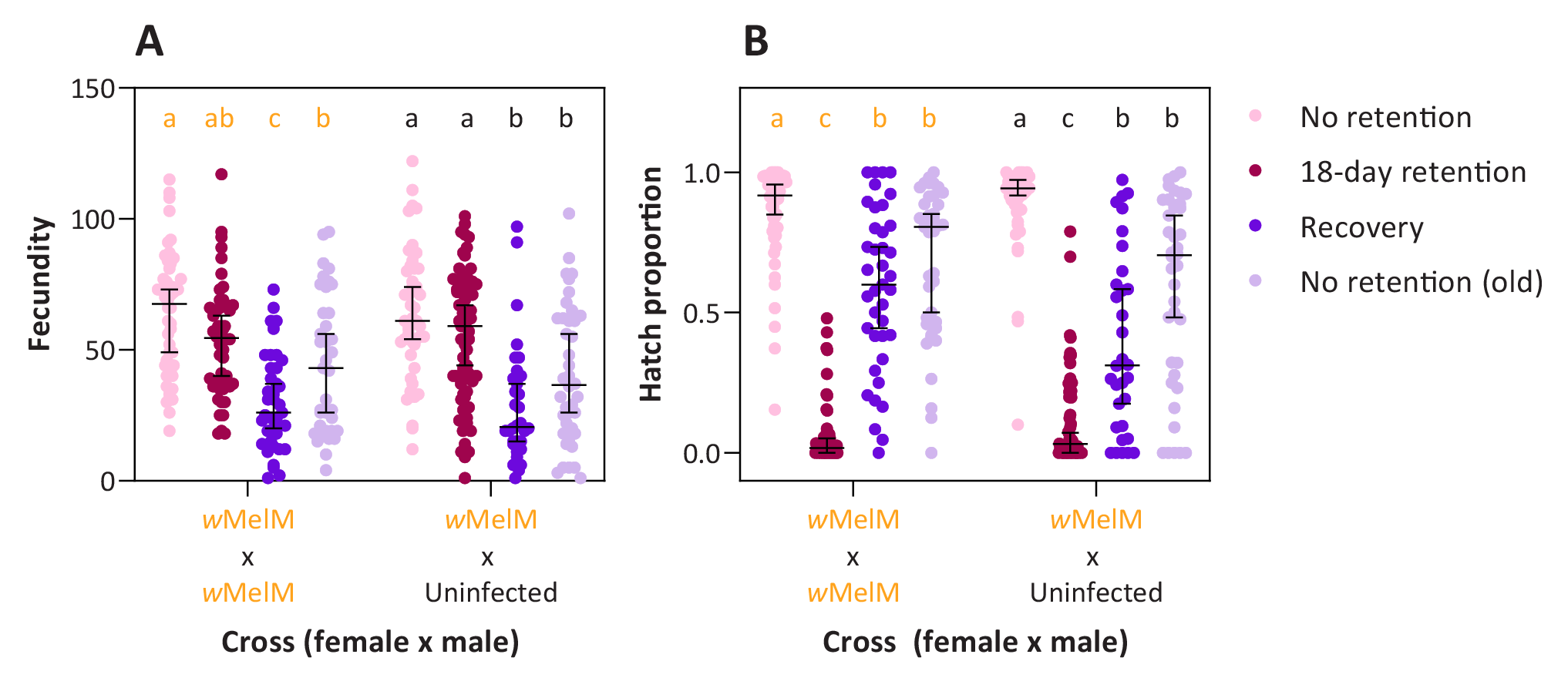


**Figure S2. Male effects of *w*MelM on the quality of retained eggs.** (A) Fecundity and (B) hatch proportions of eggs laid by *w*MelM females after mating with *w*MelM or uninfected males. Females were blood fed at 5-6 d old and experienced no retention (pink) or 18 days of egg retention (maroon). Females were also blood fed at 24-25 d old following 18 d of egg retention (dark purple) or no egg retention (light purple) for an additional gonotrophic cycle. For the full experimental design see Figure 2B. Dots show data for individual females while horizontal lines and error bars show medians and 95% confidence intervals. Within each cross, different letters represent significant differences (P < 0.05) between egg retention treatments based on Tukey’s post-hoc tests with a correction for multiple comparisons.


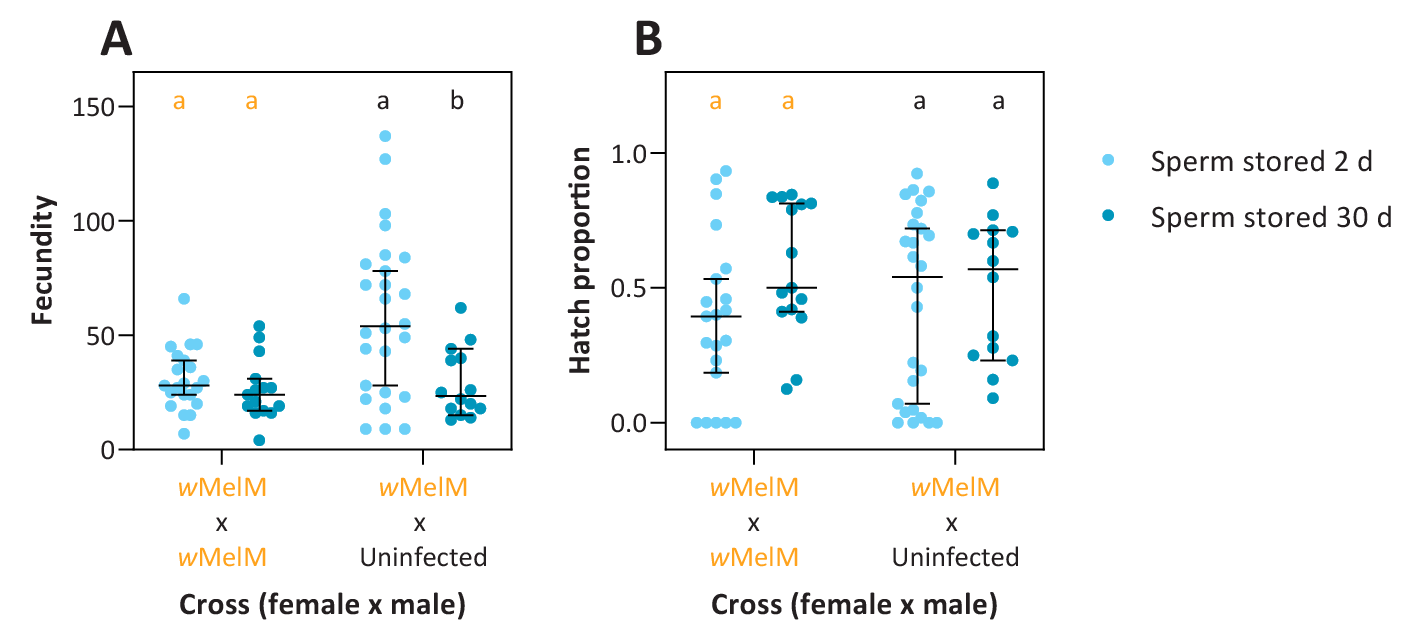


**Figure S3. Male effects of wMelM on fecundity and egg hatch following sperm storage.** (A) Fecundity and (B) hatch proportions of eggs laid by *w*MelM females after mating with *w*MelM or uninfected males. *w*MelM females were crossed to 3 d old males when they were either 3 or 31 d old, then blood fed 2 d (light blue) or 30 (dark blue) after mating respectively. For the full experimental design see Figure 2C. Dots show data for individual females while horizontal lines and error bars show medians and 95% confidence intervals. Within each cross, different letters represent significant differences (P < 0.05) between sperm storage treatments based on Tukey’s post-hoc tests with a correction for multiple comparisons.
